# Challenges in visualizing endogenous *loci* in the human genome using CRISPR-based imaging systems

**DOI:** 10.5114/bta/217939

**Published:** 2026-03-12

**Authors:** Mateusz Nowaczyk, Konrad Kuczynski, Łukasz Przybył, Marta Olejniczak

**Affiliations:** Institute of Bioorganic Chemistry, Polish Academy of Sciences, Poznan, Poland

**Keywords:** CRISPR-Cas9, CRISPR-based imaging, DNA visualization, live-cell imaging, methods

## Abstract

One of the major applications of the CRISPR-Cas9 system is the visualization of DNA by using nuclease-deactivated Cas9 (dCas9), which, following complexation with a single-guide RNA (sgRNA), specifically binds to target genomic sequences without inducing DNA breaks. In this approach, either dCas9 or the sgRNA is labeled with fluorescent proteins or dyes, or they are engineered to recruit such molecules. A key advantage of CRISPR-based imaging is that genomic elements in living cells can be tracked, because of the ability to express all system components *in vivo*. Although CRISPR-based imaging has been successfully used to label repetitive sequences in living cells, the visualization of nonrepetitive loci remains a challenging issue. The primary obstacles are a low signal-to-noise ratio and the potential for nonspecific DNA binding by the dCas9-sgRNA complex, which can generate fluorescent puncta at off-target sites. Efficient intracellular delivery of system components and their sustained expression over time are also a major concern. Consequently, CRISPR-based imaging remains a highly time- and labor-intensive process that requires ongoing optimization. Here, we summarize recent advances in labeling nonrepetitive genomic loci, outline key challenges associated with CRISPR-based imaging, and present insights derived from our own experimental findings and research experience.

## Introduction

Many essential cellular processes, including DNA replication, gene expression, DNA damage repair, and cell differentiation mechanisms, depend on the organization of genomic DNA and chromatin dynamics (Wu et al. 2019; Park and Kim 2025). Therefore, to fully understand these fundamental processes, it is crucial to advance our knowledge of the spatiotemporal behavior of chromatin within its native cellular context. Fluorescence in situ hybridization (FISH), a conventional method for DNA imaging, requires cell fixation and therefore cannot be applied to chromatin imaging in living cells, which enables tracking of DNA elements over time. To overcome this limitation, clustered regularly interspaced short palindromic repeat (CRISPR) imaging methods have been developed. Originally, the CRISPR system was applied to precise genome editing by utilizing the ability of the CRISPR-associated 9 (Cas9) nuclease to be directed by a single-guide RNA (sgRNA) to almost any genomic sequence, inducing a double-strand break at the target site (Jinek et al. 2012). Subsequently, the CRISPR-Cas9 system has been repurposed for numerous other applications, including gene expression regulation, epigenome editing, and visualization of genomic *loci* (Wang et al. 2016a). A major advantage of CRISPR-based imaging is that it enables live tracking of genomic elements, as all system components can be easily expressed *in vivo*. CRISPR-based genomic imaging methods utilize nuclease-deactivated Cas9 (dCas9), which, upon complexation with an sgRNA, can specifically bind to target sequences in the genome. To enable DNA visualization, dCas9 was initially fused to a fluorescent protein (dCas9-FP), allowing the labeling of repetitive sequences in telomeres, pericentromeric regions, and coding genes such as *MUC4*, which contains 100 to 400 tandem repeats of a 48-bp sequence in its second exon (Chen et al. 2013; Ma et al. 2015). Repetitive sequences are readily labeled using this approach, as they enable the recruitment of multiple dCas9-FP molecules to a single *locus* through a single sgRNA. Although CRISPR-based genomic imaging has huge potential, there are significant limitations in visualizing nonrepetitive sequences. In this review, we present recent advancements in the field, focusing on the labeling of nonrepetitive genomic *loci* in living cells, and highlight the challenges faced in CRISPR-based imaging experiments, together with insights from our own research experience.

### Efficiency and specificity of CRISPR-based imaging approaches

The major problem associated with DNA visualization using CRISPR-based imaging methods is the low signal-to-noise ratio (SNR). Constitutive expression of dCas9-FP results in high background signals originating from unbound dCas9-sgRNA complexes. Moreover, dCas9 fused to green fluorescent protein (dCas9-GFP) in its apo state (without an sgRNA) tends to localize to the nucleolus, likely due to its interactions with ribosomal RNA (rRNA) or nucleolar proteins (Chen et al. 2013; Chen et al. 2016). Another crucial issue in the specific visualization of *loci* of interest using CRISPR-based imaging is the tendency of dCas9-sgRNA complexes to bind to undesired genomic sites (off-targets). The dCas9-sgRNA complex can bind to DNA sequences containing several mismatches relative to the sgRNA recognition region, thereby generating fluorescent puncta at off-target *loci* (Kuscu et al. 2014; Wu et al. 2014). Therefore, it is critical to carefully design sgRNAs and optimize the number of sgRNAs and the amount of dCas9 expressed in cells to minimize off-target binding while maintaining sufficient on-target signaling.

Although the ability of CRISPR-based imaging methods to visualize repetitive sequences is well established, there are still limitations in labeling nonrepetitive *loci*(Park and Kim 2025). A previous study successfully visualized the nonrepetitive *locus* in the *MUC4* gene by using the simplest CRISPR-based imaging system exclusively involving the dCas9-GFP-sgRNA complex; however, a large set of 36 sgRNAs was required to detect all three *MUC4* alleles (Chen et al. 2013). To overcome the issue of high background signal, the sgRNA sequence was modified, which improved its stability and enhanced its assembly with dCas9, increasing the SNR by approximately 5-fold. In another approach, named CRISPRdelight, the type V CRISPR–Cas system employs endonuclease-deficient Cas12a (dCas12a) fused to FP (Yang et al. 2024). Cas12a uses considerably shorter crRNAs than Cas9, as it processes its own pre-crRNA, transcribed from the CRISPR array into mature guide RNAs. This feature was used to encode up to 48 crRNAs in an engineered CRISPR array within a single plasmid to visualize several nonrepetitive *loci*. However, at least 24 crRNAs were required to fluorescently label the desired *loci.*

### Strategies to improve CRISPR-based imaging methods

As indicated above, the visualization of nonrepetitive sequences remains difficult due to the intrinsically weak signal produced by a single dCas9-FP-sgRNA complex and the possibility of nonspecific DNA binding by dCas9-sgRNA. Therefore, there is an urgent need to design multiple sgRNAs targeting adjacent sequences (which is technically demanding and time- and labor-consuming) or to increase the number of fluorescent proteins recruited to a single dCas9-sgRNA complex.

**Figure 1:**
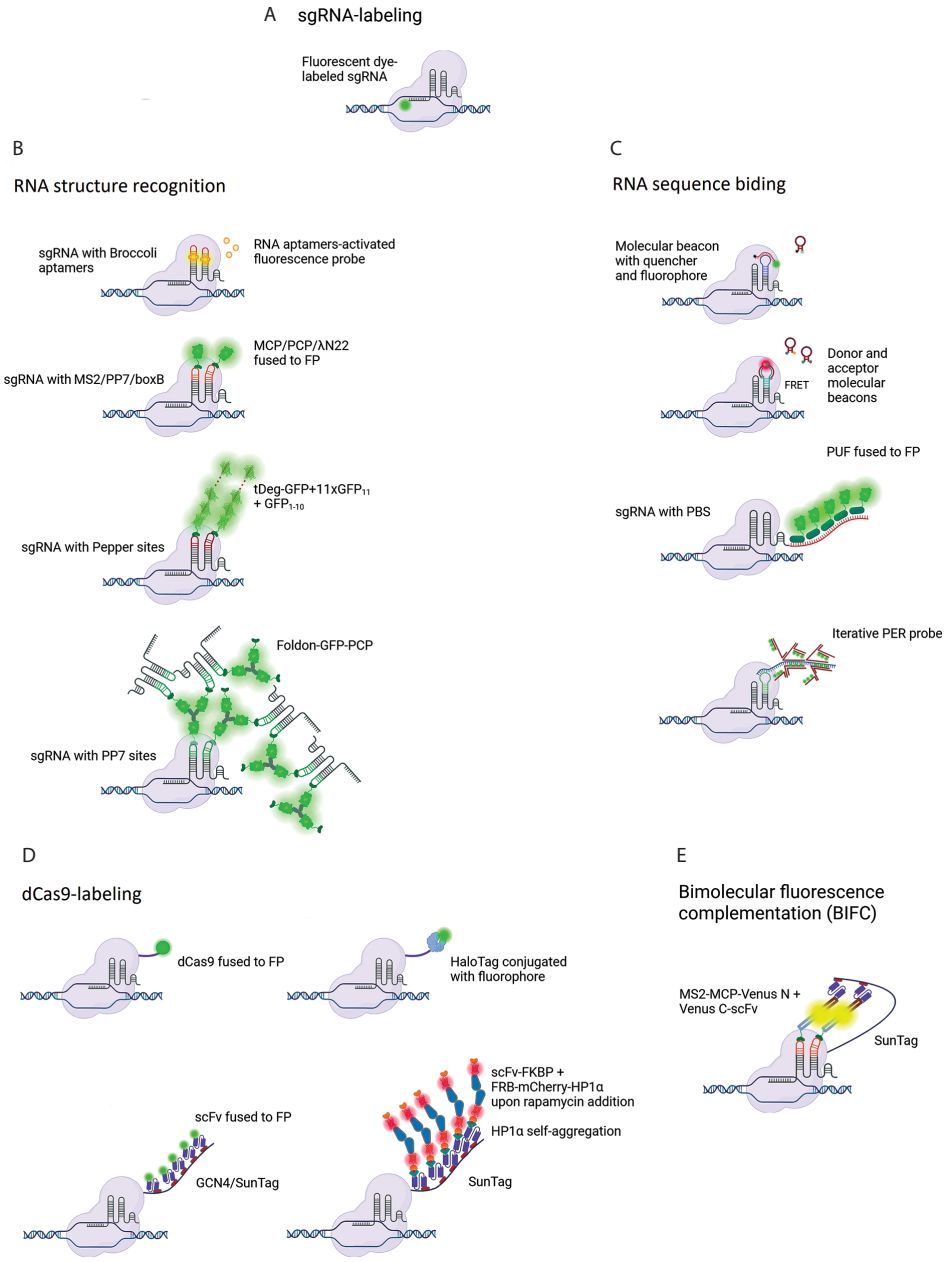
Development of CRISPR-based imaging systems. sgRNA-labeling methods include (**A**) direct labeling of the sgRNA with a fluorophore, (**B**) methods relying on RNA sequence recognition, and (**C**) methods based on the recognition of structural motifs incorporated into the sgRNA. (**D**) Methods based on dCas9 labeling. (**E**) A combined approach based on bimolecular fluorescence complementation. Created in BioRender

Attempts to increase the SNR in CRISPR-based imaging methods follow one of three strategies. The first approach is based on sgRNA labeling and includes RNA structure recognition or RNA sequence binding; the second approach is based on dCas9 labeling; and the third approach is a combination of the first and second approaches. The evolution of CRISPR-based genomic imaging methods is presented in Figure 1.

### sgRNA-labeling strategy

The direct labeling of sgRNA with a fluorophore was used in a study where the dCas9-sgRNA complex was delivered into cells through electroporation (Figure 1A) (Wang et al. 2019). In the sgRNA labeling strategy, which utilizes RNA structure recognition, an aptamer such as *Broccoli* can be introduced into the sgRNA (Figure 1B). This modified sgRNA binds to a specific small molecule (e.g., DFHBI-1T) and subsequently activates its fluorescence (Ma et al. 2016a). Another approach involves introducing special hairpins into the sgRNA, such as MS2, PP7, boxB, or Pepper motifs into the tetraloop, stem loops, or at the extended 3′-end. These motifs are specifically recognized by MCP, PCP, λN22, or Tat peptide embedded within a degron domain (tDeg), respectively, which are fused to FP (Fu et al. 2016; Ma et al. 2016b; Wang et al. 2016b; Qin et al. 2017; Shao et al. 2017; Ma et al. 2018; Lyu et al. 2022; Chen et al. 2024: 202).

**Figure 2:**
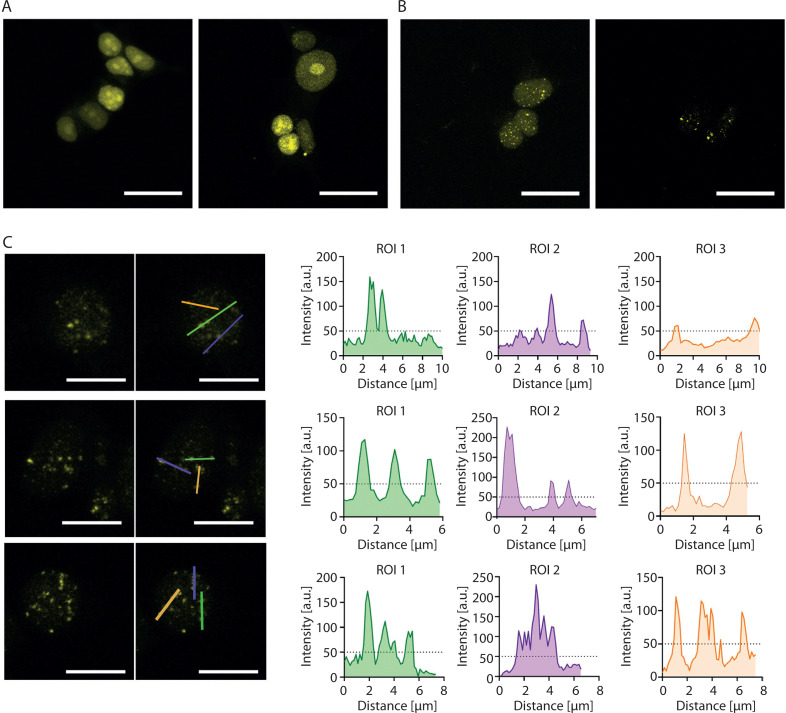
Attempts to visualize the HTT locus using the CRISPR-based imaging system. (**A**) Diffuse YFP-derived nuclear signal observed after lentiviral cocktail transduction. (**B**) Multiple YFP foci observed after co-transfection with 18 plasmids encoding 2.0 16x-MS2 sgRNAs. (**C**) Loci intensity analysis visualized using the YFP signal. Left: a fluorescence microscopy image showing the locations where the linear fluorescence profile was examined. Right: the linear fluorescence profiles corresponding to the images on the left. The analysis indicates the inability to distinguish the expected two signals from other signals in the cell. This may indicate a larger-than-expected number of loci in the studied cells. All micrographs and fluorescence profiles were obtained using Leica Application Suite X version 3.4.1.18368. All images were captured using the same parameters during a single session. Scale bar in each image: 20 µm

Although the introduction of 16 MS2 sites into the sgRNA (2.0 16x-MS2) enables visualization of a nonrepetitive *locus* with as few as four sgRNAs by using Lattice Light Sheet Microscopy (Qin et al. 2017), our attempts to visualize the *HTT locus* using 2.0 16x-MS2 sgRNAs together with yellow fluorescent protein fused to MS2 coat protein (YFP-MCP) were largely unsuccessful. When lentiviral cocktail transduction was performed as described by Chen et al. (Chen et al. 2013), no detectable YFP foci were observed (Figure 2A). Similarly, the delivery of only a few plasmids encoding sgRNAs failed to produce detectable foci, whereas the delivery of up to 18 sgRNAs yielded multiple YFP foci (Figure 2B). The number of observed YFP foci exceeded the expected number of *HTT* alleles. Consequently, *HTT* allele-derived signals could not be differentiated from nonspecific signals (Figure 2C).

These results indicate that even advanced CRISPR-based imaging approaches may generate nonspecific genomic signals and that the number of sgRNAs used must be optimized for each genomic *locus* under investigation.

Alternatively, in CRISPR-based imaging methods that utilize RNA sequence recognition (Figure 1C), researchers have introduced an sgRNA hairpin complementary to molecular beacons (MBs) carrying a fluorophore and a quencher at their ends (Wu et al. 2018) as well as used two distinct MBs capable of undergoing fluorescence resonance energy transfer (CRISPR/dual-FRET MB) (Mao et al. 2019). The sgRNA 3′-end extension with numerous PUF-binding sites (PBS), which are bound by a PUF protein fused to FP, was also used (Cheng et al. 2016; Zhang and Song 2017; Clow et al. 2022). Another method involves incorporating a short engineered sequence into the sgRNA that serves as a template for a primer exchange reaction, followed by the hybridization of numerous fluorophore-bearing imager strands, thereby amplifying the signal from the dCas9-sgRNA complex (Li et al. 2023).

### dCas9-labeling strategy

The second strategy is based on dCas9 labeling (Figure 1D) and involves fusing dCas9 directly with FP (Chen et al. 2013) or with tags that recruit many FPs to a single complex (Tanenbaum et al. 2014; Ye et al. 2017; Shao et al. 2018; Chaudhary et al. 2020; Peng et al. 2022) or labeling dCas9 with a fluorophore through a covalent HaloTag–ligand interaction (Deng et al. 2015; Knight et al. 2015; Geng and Pertsinidis 2021). Hong et al. performed an interesting comparison of dCas9-based and gRNA-based labeling strategies within the same experimental setting and cellular context (Hong et al. 2018). They found that, in contrast to the SunTag-based system, gRNA-labeling approaches generate fluorescent foci even in the absence of dCas9. These signals result from the accumulation of sgRNA transcripts near the sgRNA-expressing plasmid, potentially leading to ambiguity in the specificity of *locus* visualization. Moreover, they designed and tested several bimolecular fluorescence complementation (BIFC) strategies based on the reconstitution of fluorescent protein fragments fused to an adequate protein: scFv and/or MCP, which bind to GCN4/SunTag fused to dCas9 and MS2 sites present in the sgRNA, respectively (Figure 1E). The authors demonstrated that each designed combination has a higher SNR and does not form nonspecific foci; however, they did not test their system for nonrepetitive sequences.

### CRISPR-based imaging approaches with single sgRNA

In latest studies, more sophisticated modifications of CRISPR-based imaging systems have been developed that successfully enable visualization of nonrepetitive genomic *loci* in living cells by using even a single sgRNA (Table 1). Lyu et al. designed a DNA live imaging system termed CRISPR-mediated fluorescence in situ hybridization amplifier (CRISPR FISHer), which allows visualization of nonrepetitive DNA *loci* in live cells by using a single sgRNA (Lyu et al. 2022). In this method, an sgRNA containing two PP7 RNA aptamers is used to recruit the PP7 coat protein (PCP) fused to GFP and a T4 fibritin trimerization motif, known as the foldon (foldon-GFP-PCP). Foldon-GFP-PCP forms a trimer, which subsequently recruits additional sgRNAs, followed by another foldon-GFP-PCP recruitment, leading to an exponential increase in the signal. The authors demonstrated the specificity of CRISPR FISHer by comparing it with the CRISPR Sirius system (Ma et al. 2018). While an sgRNA containing eight PP7 hairpins failed to generate a detectable signal from the nonrepetitive *locus,* CRISPR-FISHer enabled the detection of 2–4 GFP puncta resulting from foldon-GFP-PCP accumulation.

**Table 1:** CRISPR-based genomic imaging systems enabling live cell visualization of non-repetitive genomic loci using a single sgRNA

CRISPR imaging system	Components of the system	Cell line	Target	Features	References
CRISPR FISHer	dCas9, sgRNA with two PP7 motifs, foldon, GFP-PCP	HeLa, HepG2, U2OS	PPP1R2	Co-transfection with three plasmids	Lyu et al. 2022
Casilio	dCas9 sgRNA with fifteen PUF binding sites c, Clover-PUFc	ARPE-19, HAP1, HCT116/RAD21-mAID, U2OS	MUC4, MASP1–BCL6 loop, IER5L-P–IER5L-SE loop	Co-transfection with two plasmids	Clow et al. 2022
SIMBA	dCas9-24xSunTag, FRB – mCherry-HP1α, scFv-FKBP	HEK293T	IL-1B, MUC4	All components of the system encoded in one lentiviral construct HP1α induces local chromatin changes	Peng et al. 2022
CRISPR/Pepper-tDeg	dCas9, sgRNA with two Pepper aptamers, tDeg-GFP, split GFP	HEK293T	IL1-B, MUC4	Co-transfection with two plasmids	Chen et al. 2024

Clow et al. utilized the Casilio system, previously applied to imaging of repetitive elements in telomeres (Cheng et al. 2016; Zhang and Song 2017), to visualize a nonrepetitive region in the *MUC4* gene by using only one sgRNA containing 15 PUF Binding Sites c (15xPBSc), which recruits the PUFc domain fused to the fluorescent protein Clover (Clow et al. 2022). The authors preferred using PBS sequences rather than RNA hairpins such as MS2 or PP7; this is because the insertion of up to 47 PBS copies did not affect dCas9 binding activity, whereas the addition of three or more hairpins severely impaired sgRNA expression (Cheng et al. 2016).

Another method involves fusing SunTag arrays to dCas9 (Knight et al. 2015; Ye et al. 2017). The inducible system of Simultaneous Imaging and Manipulation of genomic *loci* by Biomolecular Assemblies (SIMBA) utilizes heterochromatin protein 1 alpha (HP1α) fused to mCherry and FKBP-rapamycin binding domain (FRB), which can form biomolecular assemblies (BAs) with FKBP-scFv (Peng et al. 2022). In this system, an FKBP–scFv fusion protein, which involves combining an FK506-binding domain and a single-chain variable fragment, recognizes SunTag attached to dCas9 (through the scFv arm) and, following rapamycin addition, recruits FRB-mCherry-HP1α, which can bind to another FRB-mCherry-HP1α, leading to the formation of fluorescent aggregates and heterochromatin-like condensate at the targeted *locus*. Recruitment of several FRB-mCherry-HP1α complexes enables the visualization of a nonrepetitive *locus*; however, because HP1α modulates chromatin structure, this system inherently induces local chromatin changes that may influence experimental outcomes. Moreover, rapamycin used for induction may also cause physiological changes (Li et al. 2014; Peng et al. 2022). The authors noted that HP1α may be replaced with FUS_N_, which does not affect chromatin organization, although in this case, the repetitive exon-3 region of the human *MUC4* gene was visualized rather than a nonrepetitive *locus*. Among the newest systems, only SIMBA enables delivery of all components using a single lentiviral vector; however, this approach requires a time-consuming cell line generation process involving antibiotic selection, cell sorting, and validation of expression and functional activity of each component during experiments.

The CRISPR-based imaging system CRISPR/Pepper-tDeg is also suitable for imaging nonrepetitive sequences (Chen et al. 2024). In this method, two Pepper aptamers inserted into the sgRNA are recognized and bound by a Tat peptide fused to a degron and a fluorescent protein (FP-tDeg). The degron sequence targets unbound FP-tDeg for degradation by the ubiquitin–proteasome system; however, binding to the Pepper aptamer protects it from degradation. Unbound FP degradation significantly reduces background fluorescence, thereby improving the SNR, which was found to be 5-fold higher than that of the compared CRISPR/MS2–MCP system. Visualization of a nonrepetitive *locus* using CRISPR/Pepper-tDeg was more demanding, as it required a self-complementing split-GFP system, in which the GFP1-10 fragment is expressed separately from GFP11. Products of this split gene can reconstitute functional GFP only when they are in close proximity, as occurs during SunTag binding. The lack of fluorescence from unbound split-GFP fragments further enhances the SNR and enables the visualization of nonrepetitive *loci.*

### Delivery of CRISPR-based imaging system components

One of the most critical concerns associated with CRISPR-based genomic imaging is the delivery of system components into cells. Three main delivery approaches are employed: lentiviral transduction with vectors encoding dCas9 and sgRNAs, plasmid transfection leading to the transient expression of the system, and delivery of preassembled dCas9–sgRNA complexes through electroporation. In some cases, mixed delivery strategies for dCas9 and sgRNAs have been applied. For example, in several studies, dCas9 was constitutively expressed from a lentiviral-delivered construct, whereas sgRNAs were supplied through plasmid transfection (Knight et al. 2015; Clow et al. 2022). Lentiviral transduction is commonly used to generate stable cell lines with consistent signals from the target *locus* (Chen et al. 2013; Chen et al. 2016; Peng et al. 2022). However, it is more labor-demanding than transfection, and its efficiency largely depends on the quality of the viral particles (Thuma et al. 2023). Although a high expression level of dCas9 is essential for effective DNA imaging, excessive expression of dCas9 can lead to increased background signal and unspecific DNA binding, possibly affecting cell physiology (Geisinger and Stearns 2020; Haellman et al. 2022; Xiong et al. 2023). The labeling efficiency and SNR are also influenced by the dCas9-to-sgRNA molar ratio, which is optimal between 1 : 3 and 1 : 5 (Chen et al. 2013). In some studies, to minimize strong background fluorescence, only the basal expression of dCas9-GFP was considered, despite the presence of an inducible promoter (Chen et al. 2013; Chen et al. 2016; Qin et al. 2017). Therefore, it is crucial to tightly control the amount of delivered dCas9 to facilitate chromatin imaging without negatively affecting cellular functions (Park and Kim 2025).

Because the labeling of nonrepetitive sequences requires the simultaneous expression of numerous sgRNAs targeting adjacent sites, delivering multiple constructs emerges as a challenging aspect. Chen et al. addressed this issue by transducing cells with a lentiviral cocktail composed of up to 72 individual lentiviruses, each encoding a different sgRNA for visualization of a nonrepetitive *locus* (Chen et al. 2013). However, this delivery approach does not ensure which and how many lentiviruses ultimately integrate into a given cell, making it unsuitable for experiments that require well-controlled conditions and multiple biological replicates.

**Figure 3:**
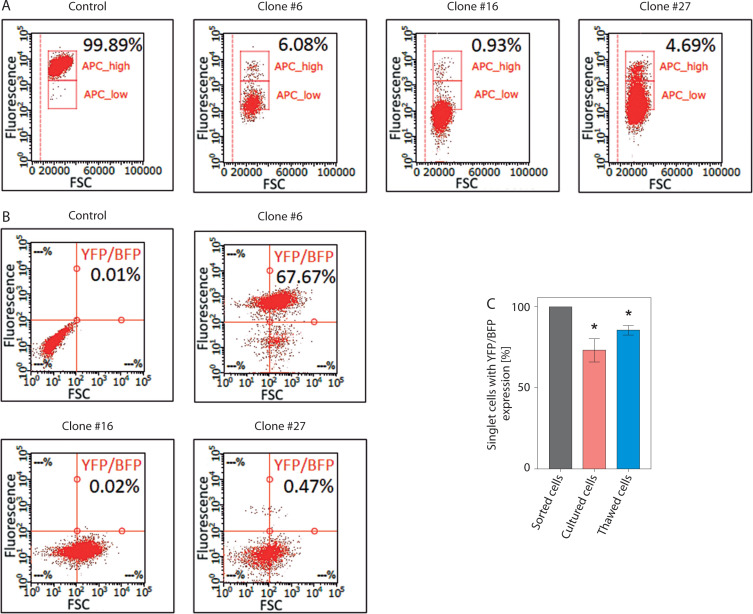
Transgene silencing during cell culture and cell banking. (**A**) Validation of dCas9-KRAB activity using a CD47 silencing assay. dCas9-KRAB, encoded together with a BFP marker, was delivered into HEK-293T cells by lentiviral transduction. After cell sorting based on BFP expression, individual clones were subjected to a CD47 silencing assay to validate dCas9-KRAB activity. The graphs show the fraction of CD47-positive cells in three HEK-293T clones expressing dCas9-KRAB following transduction with a lentiviral vector encoding an sgRNA targeting CD47. All three clones expressed functional dCas9-KRAB and exhibited approximately a 96% reduction in CD47 expression. Fractions of CD47-positive cells were determined by flow cytometry and are shown in black [%]. (**B**) Fraction of cells expressing YFP (from the construct encoding MCP fused to YFP) and BFP (from the construct encoding dCas9-KRAB) in three HEK-293T clones following cell banking and thawing. All clones were previously sorted as BFP/YFP-positive. (**C**) Fraction of cells from clone #6 expressing YFP and BFP after either thawing or following 4 weeks of culture. Cells were previously sorted as BFP/YFP-positive. Statistical analysis was performed using a one-tailed one-sample t-test; (*) indicates p < 0.05 (n = 3)

A significant issue associated with the expression of dCas9 delivered into cells using a lentiviral vector is the phenomenon of transgene silencing, which represents a major barrier to many biotechnology applications. Chromatin-modifying enzymes are recruited in both sequence-dependent and sequence-independent manners, leading to heterochromatin formation, DNA methylation, and histone modifications around the transgene *locus,* ultimately resulting in loss of transgene expression – even at frequently used safe harbor *loci* (Cabrera et al. 2022; Uenaka et al. 2025). During our experiments, we observed high frequency of transgene silencing can be common. Of three generated HEK-293T clones expressing dCas9 fused to a transcriptional repressor the Kruppel-associated box (KRAB) domain (dCas9-KRAB) (Figure 3A) and YFP-MCP from lentiviral-delivered constructs, two completely lost BFP expression (from the dCas9-KRAB construct) as well as YFP expression after cell banking and subsequent thawing (Figure 3B). The third clone showed a marked reduction in the expression levels of both reporters. Further investigation, including monitoring of BFP and YFP expression after thawing and during a 4-week culturing period, revealed a substantial decrease in both signals in cells previously sorted based on BFP/YFP expression (Figure 3C). These results emphasize the need to take adequate precautions when introducing components of CRISPR-based imaging systems through lentiviral transduction, as transgene silencing or loss of expression may impact the experimental results. Practical guidelines to mitigate transgene silencing in mammalian cells have been thoroughly reviewed by Cabrera et al. (Cabrera et al. 2022). Key factors to consider include promoter choice, use of insulators (DNA elements that act as barriers to transcriptional and epigenetic regulation of neighboring genes), genomic integration sites, and choice of cell type.

To simplify the introduction of multiple sgRNAs into cells, plasmids encoding sets of sgRNAs have been developed. By using the Golden Gate cloning method, Ma et al. generated a single plasmid encoding six sgRNAs to visualize multiple repetitive *loci* (Ma et al. 2016b). A hierarchical cloning strategy developed by Shao et al. enabled the construction of a plasmid encoding 20 sgRNAs for labeling a nonrepetitive sequence in intron 1 of the *MUC4* gene (Shao et al. 2018). In another study, the chimeric array of gRNA oligonucleotides (CARGO) was designed to express 12 sgRNAs from a single plasmid; however, co-transfection of three such CARGO plasmids was required to visualize a nonrepetitive region in mouse embryonic stem cells (Gu et al. 2018). The authors also noted that labeling efficiency varied between experimental rounds and suggested that the method would improve once a CARGO-integrated cell line was established. In a more recent study, CRISPR array encoding crRNAs for dCas12a was used, and an array of 24 crRNAs could adequately visualize a nonrepetitive *locus* (Yang et al. 2024). The authors compared their system, termed CRISPRdelight that uses plasmids encoding 48 or 67 crRNAs, with the CARGO system, involving co-transfection of three plasmids encoding 12 crRNAs each. They demonstrated that CRISPRdelight was superior in terms of the proportion of labeled cells and the SNR. Moreover, the CRISPRdelight approach was approximately 4.5-fold less expensive than the CARGO system.

The latest CRISPR-based imaging systems have been designed to use a single sgRNA per *locus* to label nonrepetitive DNA sequences. This enables rapid vector design, facilitates sgRNA delivery, and eliminates the possibility of competition among different sgRNAs for dCas9 binding. However, transfection of more than one plasmid is still required because these more complex systems rely on the expression of additional components (Clow et al. 2022; Lyu et al. 2022; Chen et al. 2024). The delivery of preassembled ribonucleoprotein (RNP) CRISPR-Cas complexes enables precise control over intracellular dCas9 and sgRNA levels (Park and Kim 2025). In some studies, RNP complexes were delivered to cells through electroporation (Wang et al. 2019), where the sgRNA was tagged with a fluorogenic dye, or by microinjection (Geng and Pertsinidis 2021), where dCas9 was fused to a fluorophore. The main advantage of using RNP delivery is that it eliminates the need for complex genetic manipulations involving multiple vector constructs. However, cells require a recovery period after the physical stress caused by these methods, and cell viability may be reduced (Park and Kim 2025). Moreover, microinjection requires specialized equipment and expertise, and it is suitable primarily for small-scale experiments.

### Adverse effects of CRISPR-based chromatin labeling

A noteworthy observation is that the interactions of the dCas9-sgRNA complex with chromatin can cause undesirable effects on cellular processes, which may impact certain experiments. Although CRISPR-based imaging methods are well established for DNA tracking, for example, in DNA repair analysis (Lyu et al. 2022; Zhang et al. 2024), DNA binding by dCas9-sgRNA can affect natural genome folding processes (Clow et al. 2022). Moreover, upon binding to DNA, the dCas9-sgRNA complex can interfere with transcription by blocking RNA polymerase progression, thereby preventing transcript elongation or hindering transcription factor binding (Qi et al. 2013). Transcriptomic analysis in human induced pluripotent stem cells revealed that dCas9 overexpression alters the expression of several genes involved in developmental processes (Haellman et al. 2022). Another study reported that dCas9 binding can locally open chromatin, thereby increasing accessibility for transcription factors in mouse embryonic stem cells and, consequently, affecting gene expression (Barkal et al. 2016). The binding of dCas9 to DNA can also hamper DNA replication and sister chromatid resolution (Xiong et al. 2023). Furthermore, dCas9 binding can induce TP53-dependent cell cycle arrest in human cells (Geisinger and Stearns 2020). These issues should be carefully considered during experimental design and subsequent data analysis.

Given that dCas9-sgRNA binding to DNA can influence chromatin organization, transcription, DNA replication, and cell cycle progression, these effects should be explicitly evaluated as part of the experimental design. For *loci* located near actively transcribed genes, it is recommended to monitor local gene expression before and after labeling, for example, by RT-qPCR or RNA-seq, to determine whether dCas9 binding interferes with RNA polymerase activity or transcription factor access. To assess potential changes in chromatin accessibility, complementary assays such as ATAC-seq or chromatin immunoprecipitation can be used to compare labeled and unlabeled cells. Simultaneously, replication-related effects can be evaluated by monitoring cell cycle profiles and DNA synthesis, for example, by using flow cytometry-based cell cycle analysis or EdU incorporation assays. Because dCas9 binding is associated with TP53-dependent cell cycle arrest, it is advisable to routinely measure cell viability, proliferation rates, and p53 pathway activation during experiments. The inclusion of dCas9-alone and nontargeting sgRNA controls can help distinguish general effects of protein overexpression from *locus*-specific effects caused by DNA binding. Finally, when interpreting imaging or functional data, results should be considered in the context of these potential perturbations. Observed changes in chromatin position, transcriptional output, or cellular behavior may reflect not only the natural state of the genome but also the local and global effects induced by dCas9 occupancy itself.

## Conclusions

CRISPR-based genomic imaging methods have been successfully applied to visualize repetitive genomic sequences in mammalian cells, providing a robust tool for tracking DNA elements in living cells. However, the visualization of nonrepetitive *loci* remains a challenging problem because of the weak signal generated by a single dCas9-sgRNA complex, tendency toward off-target binding, and the low SNR caused by the diffusion of fluorescent proteins in the nucleus. To date, several strategies have been developed to improve the performance of CRISPR imaging, including both dCas9-labeling and sgRNA-labeling approaches, and have been covered in this review. The most recent studies (Table 1) employing a single sgRNA per *locus* demonstrate substantial progress in imaging nonrepetitive DNA regions and show improvements in both specificity and SNR, confirming the great potential of these methods. However, each system still has certain limitations. The delivery of CRISPR-based imaging components typically requires the co-transfection of several plasmids, which is technically cumbersome and necessitates verification of successful introduction of all constructs. Only the SIMBA system requires a single construct delivered through a lentiviral vector; however, although this method is simple to implement, it induces HP1α aggregation at the labeled *locus,* leading to local heterochromatinization that may affect the outcomes of certain experiments. Moreover, the risk of transgene silencing is another potential limitation of methods that rely on lentiviral vector-based delivery of CRISPR-based imaging components.

Although the use of a single sgRNA for visualization represents a significant advancement over previous methods, it may still lead to off-target binding and the generation of nonspecific signals (Chen and Wang 2023). Moreover, sgRNA can bind to target DNA with different efficiency in different cell types (Thuma et al. 2023). Therefore, validation of observed fluorescent foci, such as performing FISH analysis to confirm the specificity of the system, is crucial for each genomic site intended for labeling. Additionally, adequate negative controls should be included to eliminate the possibility of nonspecific artifacts (Hong et al. 2018).

In near future, further development of CRISPR-based imaging methods is likely to focus on increasing signal specificity while minimizing biological perturbations. Novel strategies that combine fluorogenic reporters, phase separation-based signal amplification, and conditional protein stabilization show potential for achieving high SNR and reduced protein burden on the cell. Integration of CRISPR-based imaging with single-molecule tracking, super-resolution microscopy, and real-time transcriptional readouts (Hwang et al. 2024) may enable simultaneous visualization of chromatin dynamics and functional gene activity within the same living cell. Parallelly, advances in computational sgRNA design and machine learning-assisted off-target prediction are expected to optimize experimental workflows and improve reproducibility across laboratories. Ultimately, combining CRISPR-based imaging with multi-omics and high-throughput screening could transform these methods from tools used mainly to “see” DNA into robust platforms for understanding how the genome is organized and controlled, and how these processes change during development or disease.

To summarize, CRISPR-based imaging applications offer an invaluable approach for chromatin labeling and DNA tracking in living cells, and they have significant potential for further development. However, these methods remain highly time- and labor-intensive and therefore require ongoing improvements, particularly in enhancing imaging accuracy, optimizing delivery of system components, and refining sgRNA design.
